# Human Infection with M- Strain of *Brucella canis*

**DOI:** 10.3201/eid1001.020622

**Published:** 2004-01

**Authors:** Jorge C. Wallach, Guillermo H. Giambartolomei, Pablo C. Baldi, Carlos A. Fossati

**Affiliations:** * Hospital F. J. Muñiz, Buenos Aires, Argentina; † IDEHU, Universidad de Buenos Aires, Buenos Aires, Argentina; ‡ Universidad Nacional de La Plata, La Plata, Argentina

**Keywords:** *brucella canis*, M- strain, human infection, clinical picture, immune response

## Abstract

The less mucoid strain of *Brucella canis* or M- strain is used for the serologic diagnosis of canine brucellosis. While this strain is avirulent in dogs, we report the case of clinical brucellosis that developed in a laboratory worker a few days after handling live M- cells for antigen production.

*Brucella canis* is the causative agent of canine brucellosis, which causes contagious abortion, orchiepididymitis, and uveitis. Transmission to human requires close contact with infected animals or bacterial cultures. Symptomatic human infections are rare, probably because of the low virulence of *B. canis*; 31 human cases have been reported (*1*).

In contrast to other *Brucella* species, which are pathogenic for humans (*B. abortus, B. melitensis, B. suis*) and yield smooth colonies, *B. canis* colonies are naturally rough. Therefore, serologic tests that use suspensions of smooth brucellae are not useful in diagnosing *B. canis* infections (*2*). Since suspensions of wild-type *B. canis* tend to aggregate even in the absence of specific antibodies, a less mucoid variant termed M-, which does not produce autoagglutination is used for serologic diagnosis (*3*). The M- strain has reduced virulence in dogs; even high doses of this strain do not induce the typical signs of brucellosis in dogs (*4*). The pathogenic potential of the M- strain in humans remains unknown, and to the best of our knowledge, human infection by this strain has not been reported. We report a clinical and immunologic study of a human infection by the *B. canis* M- strain that shows that this strain can produce human disease similar to that produced by wild-type *B. canis*.

## Case Report

A 35-year-old male laboratory worker was referred to a physician with recurrent fever, headache, arthralgia, weakness, and constipation, which had begun 1 month before. The patient worked in a laboratory that produced antigens for diagnostic use. Three weeks before symptoms began, he had been handling a dense culture of live *B. canis* M- and was using no personal protection; the procedures were not performed in a biological safety cabinet. Moreover, the patient had attempted resuspension by repeated pipetting with his mouth. The clinical examination disclosed cervical adenomegaly, and laboratory tests indicated a mild increase of hepatic enzymes (Aspartate aminotransferase 46 μ/L and alanine aminotransferase 65 μ/L) and neutropenia. The patient reported not having close contact with dogs or other animals. Taking into account the unprotected exposure to *B. canis* M-, brucellosis was suspected, and blood samples were drawn for culture and serologic studies. Two weeks later blood cultures indicated a *Brucella* species that was later typed as *B. canis*. Conventional tests for antibodies to smooth brucellae (agglutination, complement fixation) yielded negative results. In contrast, slide agglutination for *B. canis* was strongly positive with undiluted serum and was also positive at 1:10 dilution. Serologic tests for hepatotropic viruses and *Toxoplasma gondii* were negative.

After diagnosis, a course with oral doxycycline, 100 mg twice a day for 42 days, plus parenteral gentamicin, 180 mg once a day for 10 days, was started. The patient clinically recovered, but on the last day gentamicin was administered, symptoms of the left VIII cranial nerve occurred, which resolved with flunarizine and vitamin B12 administration.

Blood cultures performed 2 weeks after antimicrobial therapy ended were negative for *B. canis*. During follow-up, the patient remained asymptomatic, his cervical adenitis resolved, and serum levels of hepatic enzymes returned to normal. On his last visit, 4 years after infection, the patient was asymptomatic. While he continues to handle *B. canis* M-, cultures are now performed under strict biological safety measures (biological safety cabinet, personal protection including goggles, gloves and mask, and autoclaving of contaminated material).

### Immunologic Studies

To assess the humoral immune response of the patient to *Brucella* antigens, the slide agglutination test with *B. canis* M- and 3 enzyme-linked immunosorbent assays (ELISA) were used. ELISA used a hot-saline extract of *B. canis* M- (HS, mainly composed of rough lipopolysaccharide [LPS] and outer membrane proteins), a preparation of cytoplasmic proteins of *B. abortus* depleted of LPS (CP [cytoplasmic proteins]) or recombinant *Brucella* lumazine synthase (RBLS), which were obtained as described previously (*5*–*7*). As shown in the Figure, antibodies to the three antigens were detected at the time of diagnosis, but antibodies to proteinaceous antigens (CP and RBLS) were negative earlier than those against HS. The slide agglutination test that used *B. canis* M- and undiluted serum was strongly positive at diagnosis and 48 days later (beginning and end of therapy, respectively), weakly positive at 103 and 150 days, and negative at 190 days and 4 years after diagnosis. Positive samples were assayed in serial dilutions starting at 1:10; only the initial sample was positive at 1:10 dilution (negative at 1:20).

The cellular immune response against CP from *Brucella* in peripheral blood mononuclear cells (PBMCS) was also evaluated. In vitro proliferation and cytokine gene expression were investigated as previously described (*8*). For blastogenesis assays, PBMCS were cultured with CP (10 μg/mL), RBLS (5 μg/mL), or phytohemagglutinin (10 μg/mL). Results were expressed as stimulation index (counts per minute of stimulated cultures divided by counts per minute of unstimulated cultures). Stimulation indices (SI) >2 were considered positive. For reverse transcription-polymerase chain reaction (RT-PCR), RNA was extracted from PBMCS cultured in the presence of CP, RBLS, or phytohemagglutinin for 24 hours. Results were expressed as fold increase over the messenger ribonucleic acid levels of cells cultured in the absence of antigen; increases >2 were considered specific.

CP and RBLS induced T-cell proliferation (SI >2) in PBMCS obtained from the patient before antimicrobial therapy (Table). PBMCS from a healthy person, which were run in parallel, showed no response to CP and RBLS (SI <2) (not shown). In addition, CP and RBLS induced a significant (p<0.001, nonparametric Mann-Withney *u* test) upregulation of interferon-gamma (IFN-γ), interleukin (IL)-2, and IL-10 transcripts only in PBMCS from the patient. No IL-4 induction was observed with PBMCS from the patient or the healthy control (not shown). The cellular immune response declined with antimicrobial treatment (Table), but CP-specific IFN-γ remained increased 55 days after therapy ended. In the last sample (obtained 250 days after therapy ended), all the parameters of the cellular immune response were normal (not shown).

**Table:** Cellular immune response in vitro to *Brucella* cytoplasmic proteins^a^

	Antigen	Lymphocyte proliferation (SI)	IL-2^b^ (fold increase)	IFN-γ^b^ (fold increase)	IL-10^b^ (fold increase)
Before therapy	BLS	4	8	3	3
	CP	3	10	7	4.5
End of therapy	BLS	1	1	2	1
	CP	2.5	6	4	2
55 days after end of therapy	BLS	1	1	1	1
	CP	1	1	3	1

^a^BLS, *Brucella* lumazine synthase; CP, cytoplasmic proteins; SI, stimulation indices; IL, interleukin; IFN, interferon. ^b^By reverse transcription-polymerase chain reaction.

## Conclusions

The main finding of our study is that the M- strain of *B. canis* can produce human disease, which was unexpected in view of the reported avirulent phenotype of this strain in dogs. To the best of our knowledge, this case is the first of human *B. canis* M- infection ever reported. The M- strain has been widely used for the diagnosis of canine brucellosis because it is less prone to autoagglutination than its wild-type counterpart (called m+). Based on the low virulence of the M- strain in dogs (*4*), the production protocol of the laboratory where this case occurred did not include bacterial inactivation or personnel protection during initial handling of cultures, which led to a prolonged exposure to a high number of viable bacteria. As the patient was the only person involved in the production of this strain, his co-workers were not tested for *B. canis* M-. Similar illness in the production plant was not reported. Because *Brucella* spp. is not usually transmitted from patients to healthy persons, the patient’s family members were not tested for *B. canis* M- infection.

The clinical manifestations in our patient were similar to those reported for human, wild-type *B. canis* infections (e.g., fever, headache, anorexia, asthenia, and adenitis). Previous studies in dogs experimentally infected with the M- strain showed that this strain does not revert to the m(+) phenotype in vivo (*4*). Our case may be analogous to cases of human illness by attenuated strains of *Brucella* species used for animal vaccination, mainly *B. melitensis* Rev-1 (*9*) and *B. abortus* S19 (*10*). Altogether, these human infections indicate that attenuation for animals does not necessarily mean immunity for humans and that biological safety measures must be followed in each case.

To assess the humoral immune response to the infection with the M- strain, antibodies against outer membrane antigens (HS) and to internal antigens (CP and *Brucella* lumazine synthase) were measured. Overall, low titers of antibodies were found by all tests, which is similar to those found for M- infections in dogs (*4*). Low antibody titers also could be related to early administration of antimicrobial therapy, as has been shown in patients infected with smooth brucellae (*11*). Antibodies to both external and internal antigens declined after antimicrobial therapy was begun and were undetectable 6 months after diagnosis (Figure). This decline, with longer persistence of antibodies to external antigens, is in agreement with our previous findings in human infections by smooth *Brucella* species (*12*).

**Figure:**
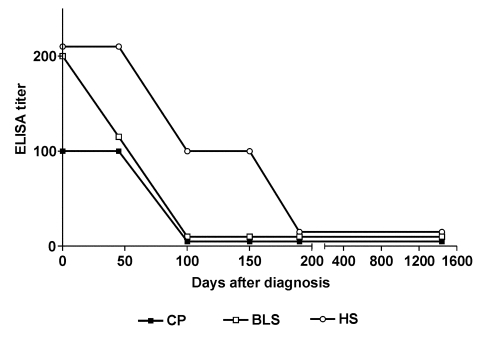
Serological follow-up of a human infection by *Brucella canis* M-. CP, cytoplasmic proteins; BLS, *Brucella* lumazine synthase; HS, *B. canis* hot-saline extract. The enzyme-linked immunosorbent assay titer was calculated as the inverse of the last serum dilution that yielded an optical density higher than the cut-off of the assay.

An early and strong cellular Th1-type response to *Brucella* internal antigens developed in this patient, in agreement with our previous observations in acute human brucellosis (*8*). The reasons for the decline of this response during follow-up are unknown, but conceivably, bacteria levels were substantially diminished by the early antimicrobial therapy, thus eliminating the internal antigens needed to develop a long-lasting cellular immune response.

In summary, this case shows that, in spite of its reduced virulence in dogs, *B. canis* M- can produce human disease with a clinical picture similar to that produced by the infection with wild-type strains of *B. canis*. Therapeutic and immunologic parameters seem to be very similar to those observed in infections by smooth brucellae.
